# Advancing healthcare through thoracic ultrasound research in older patients

**DOI:** 10.1007/s40520-023-02590-w

**Published:** 2023-11-11

**Authors:** Simone Scarlata, Chukwuma Okoye, Sonia Zotti, Fulvio Lauretani, Antonio Nouvenne, Nicoletta Cerundolo, Adriana Antonella Bruni, Monica Torrini, Alberto Finazzi, Tessa Mazzarone, Marco Lunian, Irene Zucchini, Lorenzo Maccioni, Daniela Guarino, Silvia Fabbri della Faggiola, Marco Capacci, Maria Giovanna Bianco, Guglielmo Guarona, Giuseppe Bellelli, Fabio Monzani, Agostino Virdis, Raffaele Antonelli Incalzi, Andrea Ungar, Andrea Ticinesi, On behalf of the GRETA research group on thoracic ultrasound in the older patient, Italian Society of Geriatrics and Gerontology (SIGG)

**Affiliations:** 1 Operative Research Unit of Internal Medicine, Fondazione Policlinico Universitario Campus Bio-Medico, Via Alvaro del Portillo 200, 00128 Rome, Italy; 2 Department of Medicine and Surgery, Research Unit of Geriatrics, Università Campus Bio-Medico Di Roma, Via Alvaro del Portillo 200, 00128 Rome, Italy; 3 School of Medicine and Surgery, University of Milano-Bicocca, Via Giovanni Battista Pergolesi 33, 20900 Monza, Italy; 4 Department of Neurobiology, Care Sciences and Society, Department of Geriatrics Aging Research Center, Karolinska Institutet and Stockholm University, Stockholm, Sweden; 5 Department of Medicine and Surgery, University of Parma, Via Antonio Gramsci 14, 43126 Parma, Italy; 6 Geriatric-Rehabilitation Department, Azienda Ospedaliero-Universitaria di Parma, Via Antonio Gramsci 14, 43126 Parma, Italy; 7 Acute Geriatric Unit, Fondazione IRCCS San Gerardo de Tintori, Via Giovanni Battista Pergolesi 33, 20900 Monza, Italy; 8 Geriatrics and Intensive Care Unit, Azienda Ospedaliero-Universitaria Careggi, Largo Brambilla 3, 50134 Florence, Italy; 9 Department of Clinical and Experimental Medicine, University of Pisa, Via Paradisa, 2, 56124 Pisa, Italy; 10 Department of Experimental and Clinical Medicine, University of Florence, Largo Brambilla 3, 50134 Florence, Italy; 11 Casa di Cura Venerabile Confraternita di Misericordia Navacchio, 56023 Pisa, Italy

**Keywords:** Chest ultrasound, Lung ultrasound, Diaphragm motility, Respiratory failure, Geriatric assessment

## Abstract

This paper reports the proceedings of a meeting convened by the Research Group on Thoracic Ultrasound in Older People of the Italian Society of Gerontology and Geriatrics, to discuss the current state-of-the-art of clinical research in the field of geriatric thoracic ultrasound and identify unmet research needs and potential areas of development. In the last decade, point-of-care thoracic ultrasound has entered clinical practice for diagnosis and management of several respiratory illnesses, such as bacterial and viral pneumonia, pleural effusion, acute heart failure, and pneumothorax, especially in the emergency–urgency setting. Very few studies, however, have been specifically focused on older patients with frailty and multi-morbidity, who frequently exhibit complex clinical pictures needing multidimensional evaluation. At the present state of knowledge, there is still uncertainty on the best requirements of ultrasound equipment, methodology of examination, and reporting needed to optimize the advantages of thoracic ultrasound implementation in the care of geriatric patients. Other issues regard differential diagnosis between bacterial and aspiration pneumonia, objective grading of interstitial syndrome severity, quantification and monitoring of pleural effusions and solid pleural lesions, significance of ultrasonographic assessment of post-COVID-19 sequelae, and prognostic value of assessment of diaphragmatic thickness and motility. Finally, application of remote ultrasound diagnostics in the community and nursing home setting is still poorly investigated by the current literature. Overall, the presence of several open questions on geriatric applications of thoracic ultrasound represents a strong call to implement clinical research in this field.

## Introduction

In the last decade, bedside thoracic ultrasound has become extremely popular in the hospital care of acute patients with respiratory symptoms [1]. In fact, when integrated with clinical and laboratory data, it ensures better diagnostic performances for the detection of the main causes of acute respiratory failure, including pneumonia, heart failure, pleural effusion, and exacerbation of chronic obstructive pulmonary disease (COPD), than traditional diagnostic pathways relying on chest X-rays [2]. It is also associated with quicker management and shorter boarding times in Emergency Departments, potentially improving patient outcome and reducing costs [3]. In specialist settings, the sensitivity of thoracic ultrasound for the detection of common respiratory diseases, such as pneumonia, is comparable to the one of the chest computed tomographies (CTs) [4, 5].

The diagnostic advantages of thoracic ultrasound are particularly emphasized in older patients [6]. Frailty, mobility-disability, and lack of collaboration due to cognitive symptoms can in fact compromise the quality of traditional radiographic images, but do not affect the diagnostic accuracy of thoracic ultrasound, which is performed at the patient’s bedside directly by clinicians and based on interpretation of artifacts rather than images of anatomical structures [7]. Although only few thoracic ultrasound studies have been focused on older patients to date, the advantages for clinical management are so evident that this diagnostic method has been recently proposed as a cornerstone of acute geriatric care [6].

The emergence of the coronavirus disease 2019 (COVID-19) pandemic, which has particularly hit older patients with multi-morbidity and frailty, has allowed us to explore the use of thoracic ultrasound in other settings of geriatric care, especially in nursing homes [8–10]. In fact, studies conducted in the earliest phases of the COVID-19 pandemic have suggested that thoracic ultrasound is non-inferior to chest CT in defining the presence of interstitial pneumonia, estimating its extension and predicting prognosis [11, 12]. Thus, thoracic ultrasound was empirically found by primary care and nursing home physicians as extremely useful for triaging the severity of COVID-19 and improving the appropriateness of hospitalizations during pandemic peaks [13–15].

Despite these interesting applications, the use of thoracic ultrasound remains relatively limited in geriatric settings, as suggested by a survey conducted among Italian geriatricians before the pandemic emergency [16]. Research on the applications of thoracic ultrasound in clinical scenarios of interest for geriatric medicine is also lacking [6].

Therefore, the aim of this narrative review is to identify knowledge gaps in geriatric thoracic ultrasound research and propose novel areas of development for optimizing the use of chest ultrasound in older patients and, ultimately, improving clinical care.

## Methods

The Research Group on Thoracic Ultrasound in Older Patients (Gruppo di Ricerca sull’Ecografia Toracica nell’Anziano, GRETA) of the Italian Society of Gerontology and Geriatrics was founded in 2018 with the aim of promoting knowledge and diffusion of thoracic ultrasonography in geriatric settings through educational and research initiatives. It is composed of clinicians and academicians with recognized expertise in thoracic ultrasonography, mainly of geriatric background but also with competence in emergency medicine, intensive care, and respiratory medicine. It also includes some residents in geriatric and emergency medicine with experience in thoracic ultrasonography.

This paper represents the summary of the proceedings of a meeting of the GRETA Group held in May 2023 in Florence, Italy. Each member of the group was given the task of summarizing the literature state-of-the-art on specific topics related to the use of thoracic ultrasound in various fields of geriatric medicine (Table 1), and identify knowledge gaps with possible areas of research development and improvement in clinical practice. The results were presented by junior members of the GRETA group during the meeting and discussed jointly by all members, in order to reach an expert consensus. The final remarks on each of the topics listed in Table 1 are summarized in the following sections.

**Table 1:** List of the areas of possible development of thoracic ultrasound in research and clinical practice examined by the board of the GRETA Group and treated in the manuscript

Field	Areas examined in the consensus
Methodology of examination and reporting of lung ultrasound	- Protocol of examination - Technical requirements of equipment - Methodology of description and reporting of abnormal findings
Diagnosis and monitoring of respiratory diseases typical of the older age	- Detection of parenchymal consolidations - Detection of pleural effusions - Detection of other pleural diseases - Detection of interstitial syndrome
Examinations complementary to lung ultrasound	- Diaphragmatic ultrasound
Thoracic ultrasound applications outside the hospital	- Follow-up of post-COVID-19 sequelae - Use of thoracic ultrasound in nursing homes and primary care

## Methodology of examination and reporting of thoracic ultrasound in older people

Despite thoracic ultrasound has emerged as a highly sensitive and specific tool for diagnosing lung and pleural diseases and for guiding invasive procedures [17–20], a universally accepted standardization of equipment, procedures, and reporting is still lacking. In some cases, a consensus on methodological issues was reached by specialist boards, but not centered on geriatric patients [21–23]. The use of standardized study protocols, concerning several issues ranging from technical requirements of hardware and software equipment to reporting of pathological findings, would allow us to optimize the reproducibility and the accuracy of ultrasound examination of the lung parenchyma, pleural structures and diaphragm [22, 23]. Aging of the respiratory system, in fact, widens the range of possible anatomical and functional abnormalities detected on imaging methods, as well as the heterogeneity of clinical presentation [24], potentially reducing the diagnostic power of ultrasound.

The research groups by Schmickl and Duggan have separately performed in vitro and in vivo studies to identify the optimal settings of ultrasound equipment in order to find a compromise between image quality and visualization of artifacts of diagnostic value, namely B lines, in thoracic ultrasound [25, 26]. B lines are vertical artifacts reflecting parenchymal modifications implying increased density of the peripheral lung parenchyma and partial loss of aeration [27, 28], associated with diffuse involvement of the interstitium and impairment of the alveolo-capillary exchange capacity [29], frequently found in cardiogenic and non-cardiogenic pulmonary edema, primary or secondary pulmonary fibrosis, and infections [30, 31]. The experiments by Schmickl and Daggan’s groups have analyzed ultrasound probes, presets, depth, gain, time-gain compensation, and focus position [25, 26], and their results are summarized in the recommendations listed in Table 2.

**Table 2:** Overview of the main recommendations for thoracic ultrasound methodology of examination and reporting derived from analysis of the literature

Issue	Recommendations
Technical issues	
Probe	3.5–5 MHz Convex
Depth	Between 12 and 18 cm
Gain	Between 50 and 90%
Time-gain compensation	High, especially in the deeper portions
Focus	Set on the pleural line
Preset	Specific (e.g., thorax, if available)
Methodology of examination	
Exam execution	12-field study protocol (6 fields per hemithorax: 2 anterior, 2 lateral, 2 posterior)
Reporting	
General recommendations for reporting	- Indicate the reason why the examination was performed - Indicate patient position during examination - State any technical difficulty encountered - Description of normal findings can be omitted - Reasons for incomplete assessment of thoracic fields should be provided - Use anatomical landmarks (rib spaces, anatomical lines) to describe the position of abnormal findings - Include a conclusion section, providing clinical interpretation of the visualized findings - Diagnostic hypotheses should be based on integration of ultrasound imaging with clinical and laboratory findings
Reporting of pleural abnormalities	- Absence of pleural sliding should be always reported - Any irregularity or thickening in pleural line should be always reported, specifying the affected areas
Reporting of pleural effusions	- Always indicate the patient position during assessment of effusions - Estimation of volume of effusions with validated formulas should be preferred - A semi-quantitative evaluation (minimal, moderate, extensive; count of rib spaces involved) can be also accepted - Echogenicity characteristics (cellular, septated, iso-hyperechoic, etc.) should be described
Reporting of interstitial syndrome	- Qualitative or semi-quantitative method of evaluation of B lines should be preferred over a quantitative one - Lung Ultrasound Score (LUS), consisting in grading interstitial syndrome from 0 to 4 for each thoracic field, should be reported to facilitate follow-up
Reporting of consolidations	- Quantitative measures of consolidation size is not recommended - Semi-quantitative description of consolidations (small, moderate, extensive; presence of static or dynamic air bronchogram) should be preferred

Another open issue concerns the best methodology of ultrasound scan. The recent TUONO consensus has suggested that, in an intensive care setting, the 12-field study protocol (6 fields per hemithorax: anterior superior, anterior inferior, lateral superior, lateral inferior, posterior superior, posterior inferior) represents the best compromise between examination speed and diagnostic accuracy [21]. This protocol of examination was derived from the BLUE [32] and FALLS [33] protocols, which were introduced in clinical practice more than a decade ago for the diagnostic evaluation of respiratory failure and for the hemodynamic assessment of shock, respectively.

When reporting examinations conducted with this protocol, the description of physiological findings, including pleural sliding synchronous with respiration and horizontal artifacts called A lines, could be omitted [21]. Conversely, in case of specific pathological findings, anatomical landmarks (such as rib spaces) should be indicated in the report in addition to the involved thoracic field, to facilitate reproducibility and follow-up [21]. The reason for omission of examination of one or more thoracic fields should be also included [21]. The TUONO consensus has also provided suggestions on the best way to report and describe pathological findings [21], summarized in Table 2. The use of semi-quantitative methods for grading severity of interstitial syndrome and pleural effusions is particularly encouraged for longitudinal patient monitoring [34].

The applicability of these ultrasound settings, protocols of assessment and methodology of reporting to older patients, especially those with frailty, cognitive impairment, and mobility disability, is still uncertain. More specifically, the use of standardized methods of ultrasound assessment and reporting could facilitate follow-up of acute and chronic respiratory illnesses, monitor aging of the respiratory system and, ultimately, assist clinicians in outcome predictions.

## Diagnosis and monitoring of respiratory diseases typical of the older age

### Perspectives on pulmonary consolidations in older people

Studies conducted in acute geriatric patients suggest that bedside thoracic ultrasound is superior to traditional chest radiographs, especially in terms of sensitivity, in detecting pulmonary consolidations caused by pneumonia [7, 35]. Point-of-care ultrasound is therefore already recommended as part of the usual diagnostic assessment in older patients with suspect bacterial pneumonia, shortening the initiation of an appropriate treatment, and improving the prescription of chest CT exams [6]. However, a thorough comparison of the diagnostic performance of bedside thoracic ultrasound versus chest CT is still lacking in the scientific literature, and is the object of ongoing studies, such as the OCTOPLUS trial [36].

Aspiration due to oropharyngeal dysphagia is also an important etiologic factor leading to pneumonia in older individuals [37, 38]. Aspiration pneumonia usually has a more severe clinical course than community-acquired pneumonia (CAP), and represents a major cause of morbidity and the leading cause of mortality in the long-term care and nursing homes [39–41]. In the acute care setting, the clinical presentation of aspiration pneumonia is frequently unclear and difficult to distinguish from CAP, especially if dysphagia was not documented before, although accounting for 14% of cases of pneumonia in the general population and up to 70% in subjects over 80 years old [38, 42].

According to different systematic reviews and meta-analyses, lung ultrasound (LUS) has a very good diagnostic accuracy in identifying lung parenchymal consolidations related to pneumonia in the emergency/urgency setting [2, 43]. In addition to overt consolidations with the dynamic air bronchogram sign, LUS can also identify pneumonia in earlier stages of development, as the presence of small subpleuric consolidations, irregularities of the pleural line, and focal areas of interstitial syndrome with comet-tail artifacts called B lines [44].

However, it is unclear whether there is a typical ultrasonographic pattern associated with aspiration pneumonia in older individuals, allowing us to discern aspiration from other forms of pneumonia, including CAP, healthcare-associated pneumonia (HAP), or ventilator-associated pneumonia (VAP).

To date, very few studies are available in the scientific literature with the specific aim of describing the ultrasonographic appearance of aspiration pneumonia. In one of these studies, conducted in a group of 17 dogs, a pattern similar to those present in humans with CAP (B lines, shred sign, static, and/or dynamic aerial bronchogram) was found, with a typical localization at the level of the right anterior regions, suggesting that the presence of consolidations on LUS in these regions may be a sign of aspiration pneumonia [45]. The only significant difference with human subjects concerned the higher frequency of pleural effusions detected in dogs. In another study from Japan, the authors compared LUS, chest X-ray, and chest CT findings of a group of 34 older (mean age 87.5 years old) subjects with known aspiration pneumonia [46]. LUS finding of consolidation or presence of a focal area with a least three B lines in basal regions was highly predictive of a positive chest CT [46], while normal LUS findings allowed us to rule out the presence of aspiration [47].

Despite the interest in these studies, there is still uncertainty on how LUS can help clinicians to distinguish the etiology of pneumonia in older patients. It is also unclear whether specific LUS signs can help predict the clinical course of pneumonia, either caused by aspiration or infection, because studies on this issue are limited to anecdotical reports [47, 48]. The identification of ultrasonographic signs specifically associated with aspiration could be particularly helpful in low-resource clinical settings, such as in long-term care or nursing homes, where availability of traditional radiological exams could be limited. Early recognition of aspiration phenomena in these situations could be extremely important also from an organizational perspective, accelerating the initiation of adequate treatment and, possibly, preventing hospital referral when unnecessary.

### Perspectives on pleural effusions in the older age

Thoracic ultrasound is more sensitive and specific than X-rays in detecting pleural effusions, and is able to distinguish qualitative characteristics more effectively than CT [49, 50]. Despite the use of ultrasonography for diagnosing pleural effusions is well established in clinical practice, there are still areas of uncertainty, regarding the methods for volumetric estimation and monitoring, the capacity of differentiating exudative from transudative effusions, and the possible use of contrast agents.

Various methods of quantitative estimation of pleural effusions are described in the literature [51, 52]. Multi-planar approaches are generally preferred for millimetric estimation of effusions (e.g., before thoracentesis), through application of equations calculating the effusion volume [53]. These equations consider the height and the planimetric area of the effusion, and their performance is comparable to CT [53–56]. However, these estimates are time-consuming and may result impractical for daily clinical monitoring. For these purposes, semi-quantitative techniques, such as those implying count of involved intercostal spaces, represent valid alternatives [57]. In spite of this, evidence on the utility of semi-quantitative methods or scores to predict adverse outcomes in geriatric patients, such as repeated hospital admissions, death, prolonged hospitalization, or clinical complications, is very limited.

In a recent observational prospective study conducted in 188 patients with a diagnosis of heart failure, Lindner and colleagues developed a semi-quantitative ultrasound score ranging from 0 to 4 for each hemithorax (0 = no effusion; 1 = effusion visible only in the costophrenic angle; 2 = effusion extending over the costophrenic angle without clear separation of the lung base from the diaphragm; 3 = clear separation between diaphragm and lung base at any point of the respiratory cycle; 4 = effusion occupying > 50% of the basal pleural cavity) [58]. The score showed correlation with NT-proBNP, number of B lines, and volume of effusion calculated on chest CT, but was unable to predict clinical endpoints such as the 3-month risk of re-hospitalization and death [58]. Although potentially useful for monitoring pleural effusions in complex multi-morbid patients, this score has not been tested in geriatric patients to date.

Another major challenge in ultrasonographic evaluation of pleural effusions is represented by the differential diagnosis between exudative and transudative effusions [59]. Older patients are more exposed to risks associated with pleural drainage placement, due to reduced lung function, increased vulnerability to physical stress, frequent presence of comorbidities, higher use of anticoagulants, and possible presence of skin fragility leading to a higher risk of cutaneous lesions and infections. In this context, predicting the chemical and physical characteristics of pleural effusions through ultrasound may contribute to reduce invasive diagnostic procedures. The development of scores combining qualitative and quantitative features can be useful for achieving this goal.

Recent studies have examined echogenicity of pleural effusions using quantitative methods, such as the Hypoechogenicity Index (HI) and pixel density, which appear to correlate with the chemical and the physical characteristics of pleural effusions [60]. Soni et al. created the Ultrasound Exam Score (UES), combining pixel density with ultrasound characteristics of effusions [61]. Ultrasound images of exudative effusions showed higher pixel density than transudative effusions, and a pixel density > 10 was typical only of exudative effusions [61]. Pixel density was also significantly correlated with lactate dehydrogenase (LDH) and protein levels in pleural fluid, so that a high UES implied a high probability of exudative effusions [61]. These studies, however, are limited by small sample sizes and further multicenter prospective research, especially in the geriatric setting, is needed for validation.

Novel technological applications of ultrasound assessment of pleural effusions also include the use of contrast-enhanced ultrasonography (CEUS). In a recent prospective study, the utility of CEUS in guiding pleural drainage was evaluated [62]. In comparison with empirical techniques implying B-mode ultrasound-guided lavage with saline solution, the use of CEUS resulted in improved accuracy in pleural drainage positioning. CEUS was also more accurate than chest CT in identification of fibrinous septations that can be sometimes found in subacute and chronic effusions [62]. Therefore, the application of CEUS could assist clinicians in pleural drainage positioning, allowing also greater flexibility in choice of small-caliber catheters. Geriatric patients with complex comorbid conditions and severe mobility-limitations may particularly benefit from the use of CEUS in this setting, because this technique has the potential of reducing complications such as pneumothorax or hemorrhage.

### Perspectives on the use of ultrasound for diagnosing and monitoring other pleural diseases

The European Society for Medical Oncology 2022 Clinical Practice Guidelines for diagnosis, treatment, and follow-up of malignant mesothelioma do not include thoracic ultrasound among the clinical procedures recommended for the diagnostic approach to pleural malignancies and for monitoring patients with a history of asbestos exposure [63]. Chest CT is in fact considered the diagnostic gold standard [63]. However, thoracic ultrasound allows visualization of several pleural abnormalities. The presence of at least one among pleural-based solid lesions, pleural thickening > 1 cm, nodular pleural thickening, and diaphragmatic nodules has a specificity > 95% in detection of pleural mesothelioma [64, 65]. Sensitivity, instead, is much lower (< 40%), so that a negative thoracic ultrasound exam does not allow to rule out the presence of mesothelioma in subjects with risk factors [64, 65].

Therefore, thoracic ultrasound may not be suitable for definitive diagnosis, but could play a role in the population screening of subjects at risk of mesothelioma. In a recent study, pleural abnormalities were detected on ultrasound or CT in 57% of 117 subjects with long history of asbestos exposure, and related to older age and duration of the exposure, but not to job or smoking habits [66]. Positive and negative agreement between ultrasound and CT was 66.6% and 51.8%, respectively, while ultrasound was able to identify pleural lesions not seen on CT in 47 out of 117 participants, despite the presence of bone structures, especially scapulae, does not allow a complete ultrasonographic evaluation of the entire pleural surface [66]. Further studies are needed to explore the possible role of thoracic ultrasound as a first-level diagnostic procedure in patients at risk of asbestos-related diseases.

The use of color-flow Doppler (CFD) could be also implemented as an adjunct to the traditional B-mode ultrasonography to assist the diagnostic workflow of pleural lesions. Specifically, it could be used to identify vasculature within a lesion, because pleural malignancies are often supplied by the bronchial arterial system. According to one study, CFD has a specificity of 98% and a positive likelihood ratio of 27 in differentiating malign from benign pleural lesions [67]. CFD could also assist clinicians in discriminating between small pleural effusions and pleural thickening, as they may appear similar on B-mode ultrasound or chest CT [68]. Pleural fluids generate a color flow pattern during respiratory or cardiac cycles, that can be easily visualized during CFD ultrasound as turbulent signal, the so-called “fluid-color sign” [68]. However, such approaches require further validation before becoming suitable for clinical practice.

Thoracic ultrasound can also serve as a guidance instrument when sampling suspected pleural lesions. According to a study by Sconfienza and colleagues [69], ultrasound guidance is comparable to CT guidance in terms of sample accuracy, but is associated with relevant advantages, including reduction in procedure time, lower incidence of post-procedural pneumothorax, and reduced exposure to ionizing radiation. Ultrasound-guided biopsies of pleural lesions hold a sensitivity of 97%, an accuracy of 98%, and a diagnostic yield of 94% in differentiating malignant from benign lesions [19, 70]. The advantages of ultrasound-guided invasive procedures in geriatric patients, in terms of clinical outcomes and rate of complications, remain however unknown.

### Perspectives on ultrasound interstitial syndrome in the older age

Thoracic ultrasound has already entered clinical practice in the evaluation of diseases causing interstitial syndrome, such as acute heart failure, pulmonary edema, primary or secondary pulmonary fibrosis, and COVID-19 pneumonia [30, 31]. In particular, evaluation of number, type, distribution, and pattern of B lines can help identify heart failure phenotype, grade its severity, assist therapeutic choices, and predict the duration of hospitalization [71–75]. However, the best method to assess the severity of ultrasound interstitial syndrome pattern remains uncertain.

In the last years, artificial intelligence (AI), especially machine learning (ML) and deep learning (DL) techniques, has emerged as an effective method in mining and integrating large-scale and heterogeneous medical data for clinical practice [76]. As it is already happening in other areas of medical imaging, AI is also being applied to the analysis of thoracic ultrasound data [23]. Despite the COVID-19 pandemic AI solutions for assisting imaging diagnostics of thoracic diseases have been implemented [77], only a few studies focused on the use of AI as an aid for interpreting ultrasound images in patients with interstitial syndrome, and none of them was conducted in older patients.

In clinical practice, the common approach used to detect different lung ultrasound patterns is based on the counting and visual interpretation of artifacts. This approach was made official with the release of the Lung Ultrasound Score (LUS) in 2020, consisting of grading interstitial syndrome from 0 to 4 for each thoracic field, originally conceived for assisting the diagnosis of COVID-19 pneumonia, but applicable to all patients with interstitial syndrome [78]. Although very practical and popular, this approach is user-dependent and could lead to reproducibility issues. Technical confounding factors, related to the type of probe and equipment settings, could also contribute to increase inter-operator variability [79].

Automatic quantification of B lines by AI has the potential to decrease inter-operator variability in ultrasound assessment of interstitial syndrome. Machine-assisted quantification of lung ultrasound artifacts, according to studies conducted in small groups of patients, generally performs well [80]. However, these techniques are based on post-procedural analysis of clips and frames, and not on real-time analysis. In a recent study, Short and colleagues compared visual count of B lines by experts with AI-guided automatic detection and counted on real-time images from patients with interstitial syndrome, and found a high degree of agreement between the two methods, with high intra- and inter-observer reliability [81]. The advantages of AI could be particularly emphasized in older patients, where the acquisition of ultrasound images of good quality is generally more difficult due to lack of collaboration to examination procedures and mobility-limitations.

Inter-operator variability in thoracic ultrasonography represents an issue particularly for novice operators, although the learning curve is steep and a limited number of examinations is generally considered sufficient to guarantee an appropriate evaluation of common lung patterns [82]. To date, there is no consensus in the literature about inter-observer reliability, training duration, and level of expertise needed to perform a reliable examination. Furthermore, the minimum level of ultrasound competence to define an operator as “experienced” in thoracic ultrasound is not established [83], although, according to some authors, only 25 examinations supervised by expert physicians may be sufficient [84].

Machine-learning algorithms could improve the accuracy of thoracic ultrasound assessments made by novice operators, especially regarding the quantification of interstitial syndrome severity. In a recent prospective multicenter study conducted on patients with decompensated heart failure, the quantification of B lines by an AI software on ultrasound images acquired by novice operators was compared with the quantification made by expert reviewers on the same images [85]. The correlation between AI quantification of B lines and expert semi-quantitative evaluation was moderate to fair, suggesting that AI could make thoracic ultrasound less operator-dependent and reduce the length of training period for novice operators.

Finally, AI could assist physicians in discriminating between similar ultrasonographic patterns of interstitial syndrome. In interstitial lung disease, B-line pattern is not homogenous and generally associated with irregular and thickened pleural line, whereas in heart failure, B lines generally appear more homogeneous, bright, and bilateral, with laser beam pattern and no alterations of the pleural line [86]. Thus, the analysis of these patterns may be useful to distinguish between cardiogenic and non-cardiogenic causes of interstitial syndrome, but this is not always feasible during real-time examinations, especially in older subjects [87].

Arntfield and colleagues evaluated the capacity of a convolutional neural network to discriminate between 612 lung ultrasound videos with similar appearance but different etiology (COVID-19 pneumonia, non-COVID-19 acute respiratory distress syndrome, and cardiogenic pulmonary edema) from 243 patients, in comparison with an expert evaluation by 61 physicians with at least 3 years of thoracic ultrasound experience [88]. Interestingly, AI outperformed experts in attributing images to the correct diagnosis, while a significant variability regarding the diagnosis of COVID-19 pneumonia existed between experts [88]. These results suggest that AI techniques may help improving the diagnostic performance of point-of-care ultrasound, and the advantages may be emphasized in older people, where multiple causes of lung interstitial syndrome may coexist [89], making interpretation of ultrasound images particularly challenging.

## Examinations complementary to lung ultrasound: perspectives on diaphragm ultrasound in older people

The diaphragm is the main respiratory muscle, and its dysfunction has been associated with prolonged mechanical ventilation and weaning failure [90, 91]. Recently, diaphragmatic ultrasound has been used in acutely ill patients to quickly evaluate at bedside the respiratory efficiency by dynamic evaluation of its excursion and thickness [92]. This technique has already been validated for clinical use in patients undergoing mechanical ventilation in intensive care units [93] as an aid for defining the correct timing of discontinuation and/or the possibility of extubating [94].

Furthermore, recent studies have encouraged the use of US in the evaluation of diaphragmatic dysfunction (DD) as a reliable technique during an exacerbation of chronic obstructive pulmonary disease (COPD) and in predicting the response to non-invasive ventilation (NIMV) therapy [95, 96]. In particular, a change lower than 20% in diaphragm thickening fraction during tidal volume (ΔTdi) was demonstrated as a sign of DD and was able to predict NIV failure, longer hospital stay, and higher mortality rate [97–99].

Despite muscle atrophy occurring in mechanically ventilated patients, other aspects could affect DD during an acute exacerbation of COPD (AECOPD). Some authors hypothesized that the progressive development of dynamic hyperinflation may cause a change in the geometry of the chest wall and diaphragm, with tidal breathing closer to the individual’s total lung capacity (TLC) [100]. As such, the generation of higher-pressure levels by the respiratory muscles is required to maintain tidal volume (Vt), thus exposing the diaphragm to dysfunction and, subsequently, to fatigue. In addition to lung mechanical abnormalities due to hyperinflation, the synergic mechanisms of action of unfavorable energy balance, enhanced systemic inflammation, and prolonged use of steroids, commonly occurring in advanced lung diseases, may further worsen the development of DD during severe episodes of AECOPD [101].

On the contrary, Similowsky et al. [102] showed that the strength and the inspiratory action of the diaphragm in a group of well-nourished patients with stable COPD and hyper-inflated lungs were generally better than expected based on similar measurements in normal subjects with comparable lung volumes. They hypothesized that the chronic overload imposed on the diaphragms of these patients led to a "training hypertrophy” [102].

At the current state of knowledge, no studies have investigated DD as a prognostic parameter during an AECOPD in undernourished patients with “respiratory sarcopenia”, the muscle wasting typical of advanced lung disease [103]. This syndrome is likely due to a mixture of poor nutrition, deconditioning, aging, systemic inflammation, and sometimes medications (including oral glucocorticoids) leading to an accelerated decline in functional status, increase breathing workload, and hypercapnic respiratory failure with lesser degrees of respiratory muscle weakness. In fact, sarcopenia does not only affect the upper and lower limbs, but rather involves a more generalized loss of skeletal muscle mass and strength, which includes a decline in mass and strength of the respiratory muscles as well [104].

This aspect underlines the multiplicity of causes related to DD during COPD and the need of identification of early predictors of NIMV failure, in order to assist clinicians in stratifying the risk of negative outcomes and identifying the candidates to endotracheal intubation. Thus, early evaluation of patients under NIMV with worse survival prospects related to sarcopenia throughout a practical and low-cost tool as the diaphragmatic ultrasound could be extremely useful in clinical practice. Early identification of NIMV outcome predictors in patients with COPD who are experiencing acute hypercapnic respiratory failure consequent to exacerbation or pneumonia still remains a critical issue deserving to be further investigated even with the support of diaphragmatic ultrasound.

## Thoracic ultrasound applications outside the hospital

### Ultrasound in follow-up of post-COVID-19 sequelae

Much of the knowledge on the respiratory involvement in COVID-19 derives from studies focused on the acute phase of the disease, and there are still few data available on the long-term respiratory sequelae of the infection. Subjective dyspnea and shortness of breath are among the main symptoms frequently reported in the long-COVID syndrome, but their correlation with pulmonary imaging and respiratory functional tests still remains to be evaluated [105, 106].

Starting from the assumption that high-resolution chest CT is considered the gold standard for the evaluation of persistent interstitial lung disease [107], there are many reasons in favor of the use of lung ultrasound: it is simple to use in diversified contexts and low-cost, does not expose the patient to ionizing radiation and has already demonstrated an excellent correlation with CT findings during the acute phase of illness [11, 108].

A study by Giovannetti et al., that included 38 patients previously hospitalized in a specialist respiratory Intensive Care Unit (ICU) for COVID-19, found a high degree of overlap of chest ultrasound and HRCT abnormalities of lung parenchyma on examinations performed three months after discharge [109]. More recently, Russo et al. collected data from 74 patients hospitalized for severe COVID-19 pneumonia and subsequently evaluated 6 months after discharge, showing a clear improvement in the ultrasound and CT findings compared to baseline [110]. However, a significant percentage of participants still had morphological alterations, including focal areas of interstitial syndrome corresponding to ground-glass opacities on CT, unrelated to respiratory symptoms and therefore of uncertain clinical significance [110].

Even more recently, Barbieri et al. demonstrated a clear improvement in the ultrasound and CT findings, with excellent correlation between the two methods, in 232 post-COVID patients compared to baseline [111]. Interestingly, these authors did not find any association between ultrasound/radiological findings and subjective symptoms (still present at follow-up in 39.1% of subjects) [111].

Therefore, as suggested by these studies and other expert opinions, lung ultrasound can be proposed as a reliable first-level method for the long-term follow-up of patients with previous COVID-19, reserving CT as a second-level method for specific cases [112–114]. However, there are still several open issues to be specifically investigated, such as the use of the method in long-term follow-up (> 6 months) and in subjects at high risk for long-term respiratory sequelae, including older multi-morbid patients with previous pulmonary illnesses. An evaluation of the ultrasonographic impact of different COVID-19 pandemic waves in terms of lung sequelae would be also interesting, considering the marked differences in clinical presentations of patients hospitalized in different waves [115]. Finally, it would be important to define the clinical significance of the presence of lung ultrasound abnormalities in subjects without subjective respiratory complaints, especially in the context of aging.

### Chest ultrasound in community care: perspectives on tele-ultrasonography for geriatric patients

The technique of providing health care services remotely through the use of information and communications technology (ICT) is known as telemedicine, a term first used in the 1970s. However, elementary forms of telemedicine have been in place since the invention of telephone, which was used by physicians to provide remote counseling for non-urgent cases [116, 117]. The aim of modern telemedicine is to share accurate information for diagnosis, treatment, prevention of diseases and injuries, as well as research, with health care providers located a long way off the patient’s location. Recently, telemedicine has been used also to promote both individual and community health by educating healthcare professionals [116, 117].

The massive development of ICT in the medical field and the parallel diffusion of ultrasonography in clinical practice have recently led to the development of tele-ultrasound, i.e., the use of ultrasound in conjunction with a telemedicine connection to another instructor or other means of communication [118]. Thoracic tele-ultrasound has been boosted, in particular, by the coronavirus disease 2019 (COVID-19) pandemic, in which the quick spread of SARS-CoV-2, emergence of a high number of severe cases of respiratory failure, and necessity to keep interpersonal distances have represented huge challenges for healthcare systems [119]. This has allowed clinicians and ICT scientists to design pilot studies on the execution of tele-ultrasound examinations by non-medical personnel (nurses, medical students, or even the patient himself) or robots under careful clinical guidance, with encouraging results [120–124]. In one study conducted in rural Peru, in particular, lung ultrasound was performed in COVID-19 cases by individuals without prior experience in ultrasonography, and images were interpreted remotely by radiologists, with an elevated level of accuracy in identification of pulmonary lesions [124]. Thoracic ultrasound, in fact, was proven as a reliable diagnostic tool even when performed by non-medical personnel, including nurses or medical students [125].

Despite the promising results, the implementation of thoracic tele-ultrasound in the geriatric population encounters important barriers [126]. First, older individuals living in the community may have difficulties in using electronic devices, especially in presence of visual and hearing impairments, dementia or severe frailty. Second, reliable caregivers assisting older patients during tele-ultrasound or performing the examination may not be always available. Third, providing ultrasound equipment at home may be costly and logistically challenging. Therefore, it is no surprise that ultrasonography was not listed among the main technological interventions for remote monitoring of respiratory diseases, such as COPD, in a recent systematic review and meta-analysis [127].

Thoracic tele-ultrasound may be better applied in the nursing home setting, where healthcare professionals, not necessarily of medical background, could perform the examination, delivering images remotely to expert physicians able to give a diagnostic interpretation. The use of telemedicine in geriatrics has been studied, in fact, mainly in the nursing home setting, with promising results in terms of care organization, patient outcomes, and caregiver satisfaction [128, 129].

## Conclusive remarks

Despite the established use in clinical practice for evaluation of respiratory failure and other acute respiratory illnesses, there are several open issues regarding the application of thoracic ultrasound, especially in the field of geriatric medicine. The main ones, identified by panel discussion among the members of the GRETA Group and discussed in the previous sections of this paper, are summarized in Table 3.

**Table 3:** Overview of the main open research questions and areas of development of geriatric thoracic ultrasound identified by the GRETA Group panel

Field	Open research questions on thoracic ultrasound
Technical requirements and settings	What are the best technical requirements of ultrasound machines to optimize visualization of complex pulmonary pathologies typical of older patients? What is the best technique of thoracic ultrasound examination in geriatric patients?
Modality of reporting ultrasound examinations	What is the best way to report thoracic ultrasound examinations with multiple abnormalities, typical of multi-morbid older patients? Can geriatric thoracic ultrasound benefit from the application of a standardized report form?
Diagnosis and monitoring of pulmonary consolidations	Is thoracic ultrasound in older patients able to distinguish aspiration pneumonia from conventional bacterial pneumonia, especially in patients with known dysphagia? Is the diagnostic performance of thoracic ultrasound comparable to that of high-resolution chest computed tomography (HRCT) in identification of pneumonia in geriatric patients?
Diagnosis and monitoring of pleural effusions	What is the best method to estimate severity of pleural effusions and monitor their evolution with ultrasound in older patients? Is ultrasonographic assessment of effusion echogenicity to distinguish exudate from transudate associated with reduced number of invasive pleural procedures in older patients? Is the use of contrast-enhanced ultrasonography associated with reduced complications during pleural drainage positioning in older patients?
Diagnosis and monitoring of other pleural diseases	Is thoracic ultrasound useful as a method for screening pleural lesions in older patients with high risk of asbestos-related diseases? Can Color-flow Doppler ultrasound assist the differential diagnosis between benign and malign pleural lesions in older patients? Are ultrasound-guided procedures of pleural biopsy associated with reduced frequency of complications and better clinical outcomes in older patients?
Diagnosis and monitoring of interstitial syndrome	Can artificial intelligence improve standardization of assessment of interstitial syndrome severity in older patients and assist novice ultrasound operators in the correct interpretation of images? Can artificial intelligence improve the differential diagnosis between acute heart failure and other conditions presenting with interstitial syndrome on thoracic ultrasound?
Diaphragmatic ultrasound	Is ultrasonographic assessment of diaphragmatic excursion and thickening fraction able to predict the outcome of invasive and non-invasive mechanical ventilation in geriatric patients? Is ultrasonographic assessment able to identify diaphragmatic sarcopenia in patients with COPD? Is it able to predict clinical outcomes?
Applications of thoracic ultrasound outside the hospital	Is thoracic ultrasound clinically useful to monitor the presence of post-COVID-19 sequelae in older patients? Is there any correlation with chest HRCT? What is the clinical significance of ultrasound abnormalities detected in lung parenchyma of COVID-19 survivors? Is tele-ultrasound a reliable and cost-effective method to assess respiratory diseases in older people living in the community and in nursing homes? Does chest ultrasound maintain its diagnostic accuracy when performed by non-medical personnel with remote medical consultation in geriatric patients?

Thoracic ultrasound is a modern, accurate, quick, and inexpensive technique that has a great potential of improving the care of older patients with a wide array of acute and chronic conditions. The possible frontier applications of thoracic ultrasound, listed in Table 3, imply an urgent need of boosting research in this field, in order to obtain advancement in clinical practice. By contributing to early and accurate diagnoses, guiding interventions, and facilitating a deeper understanding of thoracic pathologies, point-of-care ultrasonography empowers healthcare providers to make informed decisions that optimize patient outcomes in a personalized way. Therefore, the GRETA Group strongly supports the need of investing in research, technology, and training to harness the full potential of thoracic ultrasound and ensure its widespread integration into geriatric care.

## Data Availability

Data sharing not applicable to this article as no datasets were generated or analyzed in the present work.
